# Influence of carbohydrate ingestion on salivary immunoglobulin A following resistance exercise

**DOI:** 10.1186/1550-2783-10-14

**Published:** 2013-03-20

**Authors:** Lara A Carlson, Robert W Kenefick, Alexander J Koch

**Affiliations:** 1 University of New England, 11 Hills Beach Road Pickus Biomedical Center #209, Biddeford, ME 04005, USA; 2 US Army Research Institute of Environmental Medicine, Natick, MA, USA; 3 Lenoir-Rhyne University, Hickory, NC, USA

**Keywords:** Carbohydrate, Immunoglobulin (IgA), Cytokines, Immune response, Resistance exercise

## Abstract

**Background:**

Heavy exercise stresses immune function, however carbohydrate (CHO) supplementation has been shown to attenuate the decline in some measures of immune function after exercise. The purpose of the study was to investigate the impact of CHO supplementation on salivary immunoglobulin A (s-IgA) levels, interleukin 2 (IL-2), and interleukin 5 (IL-5), following an acute bout of resistance exercise (RE).

**Methods:**

Ten resistance trained male collegiate athletes (21±2 yr; 174±6 cm, 80±11kg, 14%±4% fat) performed RE consisting of four exercises (leg press, lat pull-downs, bench press, and leg curls). Volunteers performed four sets of 10 repetitions at 65% of 1-RM, with 2 min rest between sets for all exercises. Subjects consumed either CHO beverage (1 g•kg^-1^ body weight) or placebo (P) prior to, during, and after RE (randomized, double blind design). Saliva and venous blood were collected pre-, post- and 90 min post-exercise.

**Results:**

No change occurred in s-IgA from rest relative to osmolality or as a secretion rate (p > 0.05). IL-2 levels were unchanged by exercise in both trials (time effect p = 0.342). IL-5 was significantly (time effect p = 0.04) decreased between rest (1.55 ± 0.07 pg•ml^-1^) and 90 min post-exercise (0.96 ± 0 .11 pg•ml^-1^), with no difference between treatments (group x time effect p = 0.610). There was no time-by-treatment interaction (p < 0.05) observed between CHO and P treatments for s-IgA or IL-5.

**Conclusion:**

IL-5 decreases after RE, but s-IgA and IL-2 levels remain stable. CHO ingestion prior to-, during or following RE did not appear to alter salivary immune responses.

## Background

Acute strenuous exercise can temporarily impact components of both innate and adaptive immunity [1]. One of the first lines of defense in the innate immune system against pathogens is salivary immunoglobulin A (s-IgA). Salivary immunoglobulins are the first barrier to colonization by microorganisms causing upper respiratory tract infections (URTI) [2], and s-IgA is the predominant immunoglobulin in mucosal fluids serving to inhibit the attachment and replication of pathogens and neutralize viruses and toxins. Whereas acute bouts of moderate exercise are not implicated in mucosal immunity, prolonged high intensity endurance exercise seems to provoke alterations in the level of s-IgA [3]– [5]. In addition, low resting levels of s-IgA have been correlated with an increased risk of URTI among competitive swimmers [5] and American football players [4]. Nevertheless, several studies have reported either an increase [6] or no change [7,8] in s-IgA following exercise. The differences in reported findings among studies may be related to the differences in modes of exercise, nutritional status, and the techniques in which s-IgA levels are expressed [6].

Cytokines, components of adaptive immunity, are proteins that control inflammatory and immune responses that are secreted by several types of immune cells. Contraction of skeletal muscle has also been shown to release several plasma cytokines (myokines) into the circulation [9]. Specifically, heavy exercise produces a rapid, transient increase in cytokine production, which entails increases in both pro-inflammatory (IL-2, IL-5, IL-6, IL-8, TNFα) and anti-inflammatory (IL-1ra, IL-10) cytokines [10]. However, the majority of studies examining cytokine responses have focused on acute endurance exercise and less is known about the effects of resistance exercise on cytokines. Willoughby et al. reported that IL-6 mRNA and plasma IL-6 increased 4–6 hr post-exercise following eccentric resistance exercise in knee extensors [11]. A study by Fatouros et al. found that IL-2 increased significantly, whereas IL-1α, IL-1β, IL-6 and IL-8 did not change following 30 min of circuit resistance training [12]. However, it has been acknowledged that there is a lack of understanding regarding how acute resistance exercise impacts cytokine responses [13].

Ingestion of carbohydrate (CHO) has been shown to significantly alter the immune response to long endurance exercise, with significantly reduced recovery lymphopenia, attenuated reduction of PHA-induced lymphocyte proliferation, and attenuated increase in pro- and anti-inflammatory cytokines [14,15]. The proposed mechanism behind these differences in the immune response to endurance exercise following CHO ingestion is the inverse relationship between glucose and cortisol [16,17]. While is some studies, carbohydrate ingestion has yielded minimal or no difference in lymphocyte proliferation [18], salivary [19], plasma cytokines [19], or muscle cytokine mRNA for TNFα or IL-1β [19]. Other studies of CHO ingestion and the immune response to resistance exercise, have found decreased post-exercise leukocytosis [19], lymphocytosis [1], and attenuated decreases in mitogen-induced IL-2 and IL-5 secretion from isolated peripheral blood mononuclear cells [20]. Furthermore, Bishop et al. reported that CHO ingestion elevated saliva flow rates during 1.5 and 2 h of cycling; whereas s-IgA concentrations decreased with the CHO ingestion [21].

While significant perturbations in immunity have been documented following endurance and resistance exercise, the main mechanism behind these alterations is thought to differ between exercise modes. Specifically, long endurance exercise is thought to invoke alterations in immune parameters primarily through cortisol-mediated mechanisms. In contrast, the hormonal milieu after resistance exercise appears to favor sympathetic nervous activation rather than cortisol-mediated effects [12,18]. In addition to its effects on cortisol, carbohydrate ingestion has also been shown to blunt the rise of norepinephrine and epinephrine during exercise [22]. This may be the primary mechanism by which it has produced alterations in the immune response to exercise. Given previous findings regarding the effect of CHO on the immune response to exercise [23], the aim of our investigation was to examine the impact of acute RE on circulating interleukins (IL-2 and IL-5) and s-IgA and further to determine whether the ingestion of CHO would attenuate that response. Specifically, we hypothesized that CHO ingestion would decrease the rise in circulating cytokines and blunt the decrease in s-IgA. To date, studies regarding resistance exercise with CHO supplementation utilized either lower-body exercises such as squats or half squats [18] or ten whole body resistance exercises with lesser intensity [19]. We focused on multi-joint, paired-exercises, utilizing both the upper and lower body, to recruit a large muscle mass and induce a greater overall stress, and possibly a greater immune response so that the impact of CHO supplementation could be investigated.

## Methods

### Participants

Ten moderately trained male NCAA Division III collegiate athletes volunteered for this study. All subjects had been training for at least 5 years, with a minimum of 2 years of weight-lifting experience. Participant characteristics for both groups are presented in Table 1. All subjects gave their written informed consent to participate in this study, which was approved by the university’s institutional review board. To minimize influence on the immune system, participants in both experiments adhered to instructions before attending exercise testing to not ingest caffeine, alcohol, or anti-inflammatory medications 24 hr before testing. In addition, participants agreed to abstain for 30 days from using large doses of vitamin/mineral supplements (>100% of recommended dietary allowances) until after the third exercise session. Participants were instructed not to engage in exercise during the 24 hr before each testing session.

**Table 1:** **Participant characteristics**, ***M*** ± ***SD***

**Characteristic**	**Experiment** (***n*** = **10**)
Age (years)	21.0 ± 2.2
Height (cm)	174.3 ± 6.2
Body weight (kg)	79.6 ± 11.1
Body fat (%)	13.9 ± 3.7
1-RM leg press (kg)	313.2 ± 66.9
1-RM bench press (kg)	94.8 ± 14.5
10-RM leg curl (kg)	53.4 ± 11.0
10-RM lat pull-down (kg)	69.3 ± 8.6
Years of training	4.5 ± 1.5

Participants were excluded from the study if they had any immunocompromised condition such as an autoimmune disease (i.e., lupus, multiple sclerosis, rheumatoid arthritis, or insulin-dependent diabetes mellitus), tested positive for human immunodeficiency virus (HIV), or had been diagnosed with acquired immune deficiency syndrome (AIDS). Participants were also excluded if they were taking prescription medications, using steroids, using ergogenic supplements (e.g., creatine) for at least 1 month before testing or had indicated that they experienced high psychological stress. Before each testing session, participants who displayed any symptoms associated with URTI illness that would alter immune-cell parameters were excluded from the study.

### Procedures

#### Strength assessment

One week before testing in both experiments, measurements of baseline height, body weight, and body composition via skinfold [24]. One-repetition maximums (1-RMs) using the 1-RM testing protocol [25] were determined for the leg press (Cybex International, Medway, MA), bench press (Sorinex Exercise Equipment, Irmo, SC), and 10-RMs were determined for the latissimus dorsi pull-down (York, PA) and leg curl (Cybex). The protocol for the 10-RM test was similar to the 1-RM, but each set required 10 repetitions. Subjects were also provided with dietary examples to follow the two days prior to the resistance exercise protocol [26].

#### Dietary control

For two days prior to testing sessions, participants were required to adhere to a macronutrient diet that consisted of the following percentages of their total energy intake: 40% CHO, 30% fat, and 30% protein. An example of the macronutrient meal plan was provided to the participants at the first session. For 2 days before the testing sessions, participants adhered to macronutrient diet [26] provided, and recorded their food intake. Dietary analyses were used to verify that the composition and energy content were similar for the 2 days before the testing sessions. Diet analyses were calculated using the Nutrition III diet-analysis software by N-Square Computing (Salem, OR).

#### Resistance-exercise protocol

**Figure 1:**
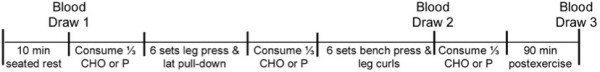
**Resistance-exercise protocol timeline.** CHO = carbohydrate; P = placebo.

The second and third testing occasions were the randomized treatment or placebo trials, which were separated by one-week. Participants were required to complete an exercise-session checklist before participation to confirm adherence to pretesting instructions. The RE timeline used for the experiment is depicted graphically in Figure 1. Participants consumed either a CHO or P beverage before, during, or after the weight-lifting session. A randomized (on first day only) double-blind treatment condition was used with the exercise protocol. PowerAde was the beverage used (8% CHO; high fructose corn syrup mixture containing 45% glucose and 55% fructose). The CHO and P beverages were designed to be identical in appearance and taste, with the CHO concentration being the only difference. The P beverage contained aspartame, citric acid, food coloring, and acesulfame potassium (a high-intensity sweetener to make the product more palatable).

On both P and CHO days of testing, participants reported to the weight room at the same time of day following a 12 hr fast. All testing took place in a 22°C environment. Participants were instructed to rest quietly in a seated position for 10 min before the first blood draw from an antecubital vein. After the first blood sample was collected, they consumed one third of a volume of fluid that contained 1 g of CHO per kilogram of body weight or an equal volume of P. After the beverage consumption and before the resistance exercise, participants stretched. Both the testing protocol and the 10-min time period after the beverage consumption were intended to prevent reactive hypoglycemia [23].

The RE protocol followed a paired-exercise format which was designed to recruit and activate a large amount of muscle tissue by having participants perform exercises that use the major muscle groups in both the upper and the lower extremities [27]. The exercise sessions consisted of exercise session consisted of two paired-exercise sets. The first paired-exercise set consisted of six sets of the leg press and six sets of latissimus dorsi pull-downs. The second set consisted of six sets of bench press and six sets of leg curls. All exercise protocols consisted of two warm-up sets of 10 repetitions of that exercise at 45% and 55% of 1-RM and four sets of 10 repetitions at 65% of 1-RM. The exercises were performed with a 2:2 cadence, and rest periods of 2 min were used between sets of exercises. The total time to complete the exercise protocol was approximately 42 min.

After the completion of the exercise protocol, a second blood sample was collected. A second dose of the same beverage and volume was administered immediately after the first paired-exercise set. A third dose of the same beverage and volume was provided after the second blood draw. At the completion of the lifting session, participants rested quietly for 90 min. The third blood sample was collected at the 90-min recovery point.

#### Saliva and blood collection and analyses

Unstimulated saliva was collected into sterile 15-ml centrifuge tubes at baseline, immediately after exercise, and at 90 min recovery. For collection, subjects were instructed to continually spit into the tubes over a timed 4 min period for a resting sample. Saliva volume was measured to the nearest 0.1 ml, and then the samples were frozen at −20°C for later analysis of IgA concentration, flow rate and osmolality. Salivary IgA concentrations were measured in triplicate (coefficient of variation (CV) = 3.1%) by enzyme linked immunosorbent (ELISA) assay. Briefly, microplates (Dynex Immulon-I) were coated with 100 μl of 2μg/ml goat anti-human IgA (Southern Biotech, #2050-01) and incubated overnight at 4°C. The following day, the plates were brought to room temperature, washed 3x with PBS (Cellgro) and blocked with 200 μl of SuperBlock (Pierce). Then the plates were washed 3x with PBS-Tween (Sigma). Saliva samples were thawed to room temperature, and then centrifuged at 1,500g for 10 min. The supernatant was diluted 1:500, added to the plates in 100 μl volumes in triplicate, and incubated for 1 h at room temperature. The plates were then washed 3x with PBS-Tween (Sigma), following which 100 μl anti-human IgA Horseradish Peroxidase (Southern Biotech, #2050-05) diluted 1:5,000 was added to the wells. The plates were again incubated for 1 h at room temperature. The plates were washed, and 100uL of substrate (Bio-Rad, #172-1067) was added to the wells. Following 30 min room temperature incubation, the plates were read on a Labsystems Multiskan MCC/340 microplate reader (Fisher Scientific, Pittsburgh, PA) at 630nm. Standards of known concentrations of purified IgA were assayed on each microplate, and absolute concentrations (μg·ml^-1^) were calculated from the standard curve. Saliva osmolality was measured in duplicate (CV = 1.3%) by a freezing point depression osmometer (Advanced Digimatic Osmometer, Advanced instruments, Needham MA).

Blood samples were drawn at baseline, immediately post-exercise, and after 90 min of recovery. All three blood samples were drawn with the participants in a seated position. Vacutainers without additive (dry) were used for interleukin (IL)-2, IL-5 levels and serum cortisol levels. Vacutainers containing sodium fluoride potassium oxalate were used for plasma lactate levels.

The blood samples for IL-2 and IL-5 were allowed to stand for 30 min after the blood draw, and then centrifuged for 10 min at 3,200 rpm. The resulting serum was frozen at −40°C and stored for later analysis. IL-2 and IL-5 were assayed by ELISA using commercially available kits (#EHIL2 and # EHIL5, Thermo Fisher Scientific, Rockford, IL) and the plates were read on a Labsystems Multiskan MCC/340 microplate reader (Fisher Scientific, Pittsburgh, PA) at 450-550nm. All samples were analyzed in duplicate (IL-2 CV = 17%, IL-5 CV = 11%). The cortisol and lactate blood samples were centrifuged for 10 min at 3,200 rpm after the blood draw, and the resulting serum and plasma was frozen at −40. Serum cortisol was assayed in triplicate using a competitive solid-phase 125I radioimmunoassay technique (Biohealth Diagnostics, Santa Monica, CA). Plasma lactate was assayed in duplicate via spectrophotometry (Sigma Kit #735, St. Louis, MO).

#### Statistical analyses

A 2 × 3 (treatment by time) repeated-measures ANOVA was used to determine whether there were significant changes in the dependent variables within a treatment or between treatments. Post hoc analyses were accomplished using paired contrasts with a Bonferroni correction. Previous studies of endurance athletes [23] have reported attenuation of immune responses of up to 25–50% with CHO supplementation. Based on this observation, we assumed that a similar change could be expected in the current study and would be considered meaningful. From Vu Tran (1997), we estimated that 6–12 participants would provide sufficient statistical power (β = 0.20) and an alpha of 0.05 to detect a difference in immune responses.

## Results

In the 2-day diet analysis before each time trial, no differences (p > 0 .05) were found for kJ/day, percent CHO, percent fat, or percent protein consumed. The participant averages for all trials were 10,088 ± 2,268 kJ/day, 46% ± 8.8%, 25% ± 3%, and 29% ± 5% for CHO, protein, and fat, respectively. Total volume (weight • sets • reps) completed during the CHO and P exercise sessions was also not different and averaged 118,239 ± 19,199 kg.

### Plasma lactate and cortisol responses

There were no significant differences between treatments with plasma lactate responses; however, a significant main effect for time (p < 0.05) observed for plasma lactate. Immediately post-exercise plasma lactate values were elevated (p < 0.05) above pre-exercise values. By 90 min post-exercise, plasma lactate values were lower (p < 0.05) than immediately post-exercise but were greater (p < 0.05) than they had been pre-exercise. No significant differences (p < 0.05) in cortisol were observed between time periods or beverages.

### Salivary IgA responses

There was no effect of CHO ingestion on IgA:osmolality (treatment x time interaction p = 0.293) or IgA secretion rate (treatment x time interaction *p* = 0.821; Table 2). No changes in IgA levels from resting values were found when considered relative to osmolality (time effect p = 0.747) or as a secretion rate (time effect p = 0.792).

**Table 2:** **Salivary immunoglobulin A responses to resistance exercise with carbohydrate ingestion or placebo** (**n**=**10**)

**Variable**	**Condition**	**Pre**	**Post**	**60min Recovery**
S-IgA secretion PLC	PLC	208.3 ± 123.5	223.7 ± 299.6	211.2 ± 148.0
rate (μg·min^-1^)	CHO	193.7 ± 92.9	189.3 ± 230.4	270.0 ± 386.1
S-IgA:osmolality	PLC	8.60 ± 5.33	13.33 ± 7.42	10.79 ± 7.84
(μg·kg^-1^)	CHO	11.00 ± 8.68	9.23 ± 7.60	10.44 ± 8.00

### Interleukin 2 and interleukin 5 responses

Resting IL-2 was significantly higher in CHO than in P (p = 0.028; Table 3). Therefore, resting IL-2 measures were entered as a covariate in a 2x2 (treatments x time) repeated measures ANCOVA. Using this comparison, IL-2 was unchanged after RE (time effect p = 0.359). There were no differences between CHO or P in IL-5 (treatment x time interaction p = 0.610). IL-5 was significantly decreased after RE (time effect p = 0.040). Specifically, IL-5 was significantly (−37%) lower than resting levels at 90 min post (p = 0.008).

**Table 3:** **Interleukin**-**2 and interleukin**-**5 response to resistance exercise with carbohydrate ingestion or placebo** (**n**=**7**)

**Variable**	**Condition**	**Pre**	**Post**	**60min Recovery**
Interleukin 2	PLC	4.62 ± 6.42*	6.14 ± 12.32	20.88 ± 29.63
(pg·ml^-1^)	CHO	64.04 ± 54.52*	36.89 ± 18.82	11.63 ± 9.90
Interleukin 5	PLC	1.73 ± 0.61	1.07 ± 0.38	0.60 ± 0.70
(pg·ml^-1^)	CHO	1.67 ± 0.32	1.43 ± 0.30	1.09 ± 0.47

*indicates p<0.01 difference between conditions.

## Discussion

Despite the tremendous growth of investigations regarding the impact of endurance exercise on immune parameters, still less is known about the effects of resistance exercise. Several investigations suggest that reduced levels of S-IgA are associated with an increased risk of URTI during periods of heavy training, and it has been suggested that CHO supplementation may influence immune indices in response to heavy exertion. The purpose of this investigation was to determine whether carbohydrate ingestion prior to-, during and following RE would alter the immune response to RE. Ours was the first study to examine s-IgA and cytokine responses using paired-exercises, which lasted over 30 min, shown to elicit a greater stress and immune response [18]. We hypothesized that CHO ingestion would result in a lesser perturbation in s-IgA and circulating cytokines from resting values as compared to placebo. The major findings of this study were: 1) resistance exercise did not result in measureable changes in s-IgA or IL-2 responses; 2) a significant reduction in IL-5 responses were observed; 3) contrary to our hypothesis, CHO supplementation prior to-, during, and following RE had no effect on immune responses. These findings help to clarify what has been previously unknown in this area.

The central premise behind our hypothesis was that carbohydrate ingestion would blunt the rise of epinephrine and norepinephrine during RE, and thus alter s-IgA and circulating cytokines measured as compared to control. Some previous studies [22] of carbohydrate ingestion during exercise have found significant reductions in epinephrine and norepinephrine while others have found no effect [28]. Thus the impact of carbohydrate ingestion on the catecholamine response to exercise appears to be variable. This variability may be explained in part by training status, with less-conditioned subjects more likely to experience a difference in the catecholamine response to exercise after carbohydrate ingestion [29]. Subjects in the present study were highly trained, RE athletes and as such may have been less impacted by the RE protocol used such that their catecholamine responses were minimal, thus CHO supplementation was not beneficial. We did not measure catecholamines in the present study but blood/plasma lactate has been cited as a proxy measure for epinephrine [30]. The lack of difference in plasma lactate between treatments in the current study could be indicative of a similar catecholamine response between the CHO and placebo conditions. It should be noted, however, that untrained individuals would likely have a greater stress and immune response from RE, especially of this intensity and duration [31] and could potentially benefit from CHO supplementation.

### IgA

Several studies have found that heavy exercise can elicit a post-exercise decrease in salivary IgA levels [32,33]. Suggested mechanisms behind an exercise-induced decrease in salivary IgA include changes in the transport of IgA across the mucosal epithelium or sympathetically-mediated vasoconstriction in the oral submucosa and consequent reduction in the migration of cells synthesizing and secreting IgA [34]. However, this finding is not consistent as other studies have reported either no change [35] or an increase [36] in post-exercise s-IgA. A likely explanation for these discrepant findings is the debate over the best method of expressing salivary IgA changes during exercise. Raw IgA concentrations do not account for changes in saliva composition typically associated with exercise [37]. IgA:Protein has been the traditional method to correct for the drying effects of exercise on oral surfaces [38]. However, exercise typically produces an increase in the total protein content of saliva, thus apparent decreases in salivary IgA:Protein following exercise may reflect changes in the total protein content of the saliva sample, rather than fluctuations in IgA [34,38]. Reflective of this confusion, the three available studies on the effects of resistance exercise on salivary IgA have reported a decrease in salivary IgA expressed relative to total salivary protein [19], no change [39] or an increase [40] in raw salivary IgA. In the present study, we observed no changes in IgA (expressed as either a flow rate or relative to osmolality). Our findings taken with those previously reported in the literature raises questions about the utility of post-exercise fluctuations in IgA. Studies that have reported a link between salivary IgA levels and URTI incidence were obtained from resting samples [5]. Transient fluctuations in post-exercise salivary IgA (not observed in the case of this study) have yet to display any clinical relevance.

### Cytokines

Previous studies have found that acute resistance exercise increases both pro-inflammatory (IL-6, IL-8, IL-10) [11,19,41] and anti-inflammatory (IL-1ra, IL-2) cytokines [12,19]. Chan et al. documented decreases in mitogen (PHA)-stimulated IL-2 and IL-5 isolated peripheral blood mononuclear cells following resistance exercise [20]. We found plasma IL-5 significantly decreased at 90 min post exercise, and IL-2 was unchanged. These findings are puzzling, but may be explained in part by alterations in circulating cell numbers. IL-2 is secreted by T-Helper 1 (TH_1_) cells, IL-5 is secreted by T-Helper 2 (TH_2_) cells and Mast cells. Resistance exercise induces fluctuations in circulating immune cells, in particular, a reduction of lymphocytes (including both TH_1_ and TH_2_) cells in the 30 min-6 h recovery period after exercise [18]. Thus the modest reduction in plasma IL-5 may simply reflect fewer circulating TH_2_ cells at that time. Additionally, [12] found only a mild inflammatory response in untrained subjects following resistance exercise (in a circuit fashion) solely on ten Universal cable machines; however, the subjects only performed 30 min of total exercise. Koch et al. suggested that the resistance exercise protocol be longer in duration, so our current study increased the duration of the resistance exercise from their 15 min protocol to 42 min [18]. As there are different types of muscle actions (i.e., isometric, isokinetic, concentric, eccentric), its been reported that exercises involving eccentric muscle contractions may induce greater muscular damage and thus a concomitant inflammatory response, which would include increased cytokine production [13]. We addressed equal time for concentric and eccentric muscle actions by having the subjects perform the exercises with a 2:2 cadence. Also, in our study, we utilized resistance-trained athletes who performed exercises designed to be similar to that used in more typical athletic regimens and recruit and activate a large amount of muscle tissue. Despite the fact that RE trained athletes participated in the present study utilizing a whole body RE protocol, we did not observe changes in IL-2 and therefore a benefit from CHO supplementation.

## Conclusions

In conclusion, this was the first study to report salivary immune responses using paired-exercises during an acute resistance training session. The paired-exercise format increased the acute exercise session duration to over 40 min in order to elicit a greater stress and immune response. The results of the present study suggest that IL-5 decreases after RE, but s-IgA and IL-2 levels remain stable. Furthermore, the present data suggest that CHO supplementation prior to-, during or following RE did not appear to alter salivary or cytokine immune responses. These findings are important, because as previously reported in the literature, CHO supplementation may assist in reducing exercise-induced suppression of various aspects of the immune system. However, it appears that in highly resistance exercise trained athletes, IL-2 or s-IgA were not altered by an acute bout of heavy RE, nor were immune responses impacted upon by CHO ingestion. It is important to note that these volunteers had numerous years of RE training experience and their immune function could be adapted to such heavy RE bouts. It remains unclear whether novice resistance exercise individuals who are less adapted to the stressful insult to the body, may experience a greater degree of inflammation and immune responses, and therefore may benefit from CHO supplementation. Based on the findings in the present investigation, it appears that carbohydrate supplementation has minimal impact on the immune response to paired resistance exercise training.

## Competing interests

The authors declare that they have no competing interests.

## Authors’ contributions

LAC designed the study, secured funding, and was involved in the data collection and analysis, as well as manuscript preparation. RWK assisted with both data and statistical analyses, and manuscript development. AJK provided assay support, statistical and data analyses, and assisted with manuscript preparation. All authors read and approved the final manuscript.
